# Immune-Related Prognostic Model in Colon Cancer: A Gene Expression-Based Study

**DOI:** 10.3389/fgene.2020.00401

**Published:** 2020-05-08

**Authors:** Haojie Yang, Wei Jin, Hua Liu, Dan Gan, Can Cui, Changpeng Han, Zhenyi Wang

**Affiliations:** Department of Coloproctology, Yueyang Hospital of Integrated Traditional Chinese and Western Medicine, Shanghai University of Traditional Chinese Medicine, Shanghai, China

**Keywords:** colon cancer, bioinformatics, immune-related gene, immune cell infiltration, prognostic model

## Abstract

Mounting evidence supports that the malignant phenotypes of cancers are defined not only by the intrinsic activity of tumor cells but also by immune cells that are recruited and activated in tumor-related microenvironment. Here, we developed a diagnostic and prognostic model for colon cancer, based on expression profiles of immune-related genes and immune cell component. As a result, we found that B cell infiltration ratio, CD4^+^ T cells, as well as immune-related genes of *TRIB3*, *CHGA*, *CASP7*, *LGALS4*, *LEP*, *NOX4*, *IL17A*, and *HSPD1* may be highly relevant with clinical outcome of colon cancer.

## Introduction

Colon cancer is the third most common type of cancer, making up ∼10% of all cases (Forman et al., 2014), especially in developed countries, where more than 65% of cases have been found. It was reported that ∼145,290 new cases of colorectal cancer were diagnosed every year in the United States (Kim et al., 2018). In spite of the advances in screening, diagnosis, and curative resection, colon cancer is still one of the leading causes of cancer death worldwide, with unsatisfactory clinical outcome (Siegel et al., 2017). Presently, the molecular pathogenesis of this cancer is still poorly understood.

Recently, mounting evidence supports that the malignant phenotypes of cancers are defined not only by the intrinsic activity of tumor cells but also by immune cells that are recruited and activated in tumor-related microenvironment (Ben-Baruch, 2002). A previous study reported that tumor can be viewed as distinct immunological organ, which has complex immune microenvironment (Fridman et al., 2012). Pathologists have long recognized the diversity of immune infiltration into tumors, and the most widely studied are tumor-infiltrating lymphocytes (TILs) (Bense et al., 2017). TILs have been suggested to promote or inhibit tumor growth actively and are important to the clinical outcome (Brockhoff et al., 2018; Shibutani et al., 2018; Udall et al., 2018). In terms of colon cancer, study has demonstrated that the level of lymphocyte infiltration into primary tumor is a strong independent predictor of overall survival (OS). What is more, high lymphocyte infiltration represents a positive prognostic factor (Galon et al., 2006). In addition to immune cells, the cancer tissues also include various chemokines, cytokines, and growth factors (Bremnes et al., 2011). They interact with each other to form tumor-related microenvironment and exert inhibitory effects on tumor cells. Tosolini et al. (2011) have applied expression profiling of colorectal cancer to define the relevance of specific immune signatures and found that T helper 17 (Th17) type, interleukin (IL)-17-dominant immune profiles indicated a poor prognosis, and Th1 type, interferon-γ-dominant immune profiles indicated an improved prognosis.

In this study, we developed a molecular classifier associated with colon cancer prognosis based on the gene expression profiles of immune-related genes. This study may provide insights into the complex relationship between the heterogeneity of immune cells and disease prognosis in colon cancer.

## Results

### Differential Expression Analysis

**FIGURE 1:**
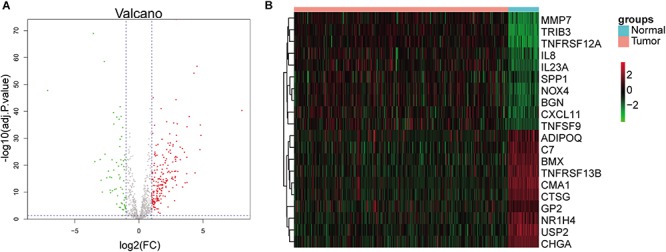
Identification of differentially expressed immune genes. **(A)** Volcano plots of differentially expressed immune genes. Red dots represent upregulated genes, and green dots represent downregulated genes. **(B)** Heatmap of top 10 up- and downregulated immune genes.

On the basis of adj. *p*-value < 0.05 and |logFC| > 1, we identified 265 differentially expressed (200 upregulated and 65 downregulated) immune genes. The volcano plot of all differentially expressed immune genes and the heatmap of top 10 up- and downregulated immune genes are shown in Figure 1.

### EPIC Immune Cell Infiltration Ratio

**FIGURE 2:**
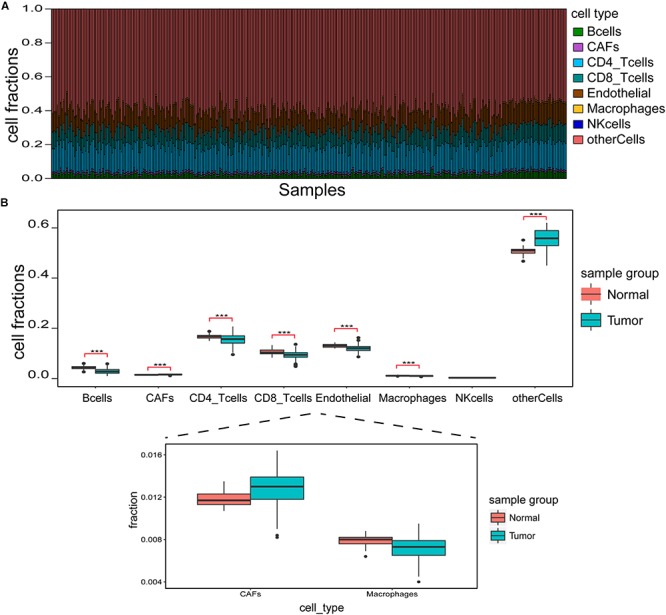
Identification of immune cell infiltration features. **(A)** Immune cell infiltration ratio in each sample. **(B)** Differences in different immune cell components between tumor and normal samples. ***p < 0.001.

Figure 2A shows infiltration ratio of immune cells. Other cells, including endothelial cells, CD4^+^ T cells, and CD8+ T cells accounted for a relatively large proportion, while B cells, carcinoma-associated fibroblasts (CAFs), macrophages, and natural killer (NK) cells accounted for a relatively small proportion. For the infiltration ratio of each immune cell, Welch two-sample non-paired *t*-test was used to conduct statistical analysis of tumor vs. normal samples, and the results showed that there were significant differences (*p* < 0.001) except for NK cells (Figure 2B).

### Diagnostic Model of Colon Cancer Based on Immune Characteristics

**FIGURE 3:**
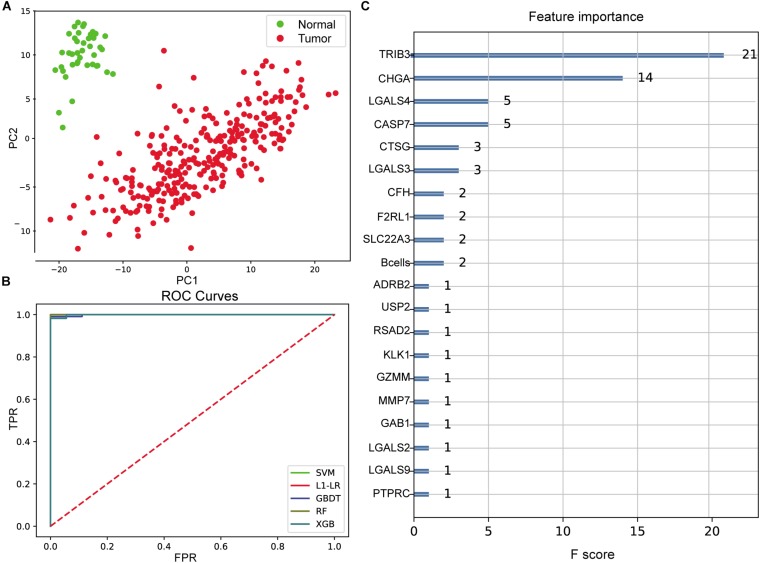
Evaluation of colon cancer diagnostic model. **(A)** Principal component analysis (PCA) of two-dimensional visualization results. PC1 and PC2 represent the first two principal components with the largest variance in PCA results, respectively. **(B)** Receiver operating characteristic (ROC) curves of model. FPR represents false positive rate [FP/(FP + TN)], and TPR represents true positive rate [TP/(TP + FN)]. The greater the area between ROC curve and *X*-axis (area under the curve value, between 0 and 1), the better the model effect. Different models are represented by different color curves. **(C)** The 20 most important features evaluated by XGB model. *F* score means the importance of the feature (the larger *F* score means the greater contribution of the feature to the model).

With immune cells component and the expression profile of differentially expressed immune genes being features for model input, the integrated sample number was 329 (288 tumor and 41 normal), and the feature number was 272 (265 differentially expressed immune genes and the composition ratio of seven immune cells except for NK cells). Dimensionality reduction in principal component analysis (PCA) was performed on the feature dimensions of each sample, and the results showed that the tumor and normal samples were significantly distinguished when only the first two principal components were used (Figure 3A), indicating that these features could be well used to construct a diagnostic model.

After cross-validation of the training model, we obtained the optimal parameters of different models and the evaluation results on the test set, as shown in Table 1. These models were excellent predictors of colon cancer samples, both in terms of accuracy and area under the curve (AUC) (the AUC values of SVM, L1–LR, and RandomForest were 1) (Figure 3B). Through the XGBoost model, we ranked the importance of features and selected the top 20 features (Figure 3C), including *TRIB3*, *CHGA*, multiple LGALS family genes (*LGALS4*, *LGALS3*, *LGALS2*, and *LGALS9*), CASP7, B cell infiltration ratio, etc.

**TABLE 1:** The optimal parameters of different models and the evaluation results on the test set.

**Model**	**Parameter**	**Accuracy**	**Area under the curve**
SVM	Kernel = “rbf;” C = 1.0; degree = 3; cache_size = 200	0.992	1.0
L1-LogisticRegression	C = 1.0; penalty = “11;” max_iter = 100; tol = 0.0001	1.0	1.0
GBDT	n_estimators = 50; learning_rate = 0.1; max_depth = 3; subsample = 0.7; min_samples_split = 3	0.985	0.996
RandomForest	n_estimators = 50; min_samples_leaf = 2; min_samples_split = 3	1.0	1.0
XGBoost	max_depth = 3; min_child_weight = 1; gamma = 0.01; learning_rate = 0.1; n_estimators = 50	0.984	0.995

### Immune Gene Expression, Immune Cell Component, and Survival Analysis

**FIGURE 4:**
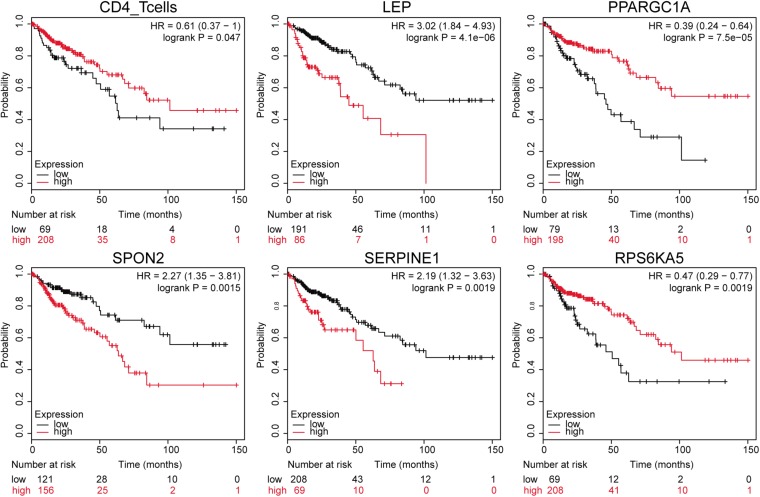
Gene expression-related survival curve. The black and red curves represent the low- and the high-expression groups, respectively.

The downloaded clinical TCGA data contained phenotypic information of 551 samples, and 277 samples had both gene expression profile and effective survival information. We extracted survival status and survival time of these samples and plotted K–M survival curve by combining the expression of 265 differentially expressed immune genes in different samples, immune cell component, and sample clinical information. The significance threshold was set as *p* < 0.05. The results showed that CD4^+^ T cells and 72 immune genes were highly relevant to prognosis. Survival curves of CD4^+^ T cells and the top five immune genes are shown in Figure 4 based on the significance of *p*-value.

### Clinical Phenotype and Survival Analysis

**FIGURE 5:**
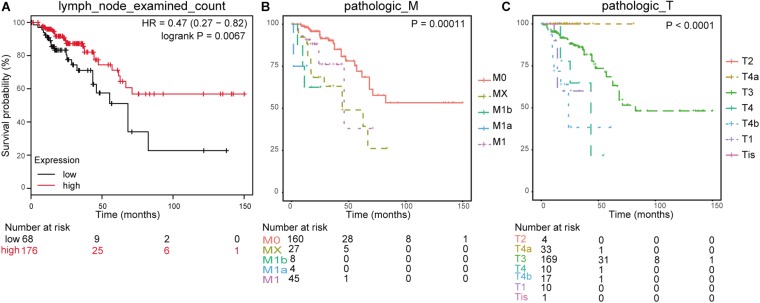
Clinical phenotype-related survival curve. **(A)** Survival analysis for lymph node examined count. **(B)** Survival analysis for pathologic_M. **(C)** Survival analysis for pathologic_T.

In line with the survival status and survival time of 551 samples in TCGA clinical data, we drew K–M survival curves in combination with gender, age, histological type, number of lymph nodes, tumor, node, and metastasis (TNM) stage, colonic polyp, and prognosis information (a total of 244 valid samples). The significance threshold was set as *p* < 0.01. The results showed that the number of lymph nodes (lymph node examined count, *p* = 0.0067), M staging (pathologic M, *p* = 0.00011), and T staging (pathologic T, *p* < 0.0001) were connected with survival time (Figure 5).

### Prognostic Model of Colon Cancer Based on Immune Characteristics

Using “train_test_split” package in Python, we divided 277 samples with both gene expression profile and effective survival information into training set and test set at a ratio of 5:5 (random state = 123). Considering the content of CD4^+^ T cells and the expression values of 72 immune genes, we used Cox multivariate regression to construct a prognostic model. The results of both likelihood ratio test and score (log-rank) test were significant, and the *p*-values were *p* = 1.493*e*−08 and *p* = 0.001621, indicating that this multivariate model was highly relevant to the prognosis. In Cox multivariate model, there were 19 factors (*p* < 0.05) of great influence, including CD4^+^ T cells, *LEP*, *NOX4*, *RETNLB*, *LAIR1, IL17A*, *HSPD1*, *CYTIP*, *SLAMF7*, *CD14*, *C7*, *CORO2A*, *PPARGC1B*, *LTB4R*, *CHGA*, *CD300A*, *TLR6*, *CD209*, and *P2RY14*.

The risk model was built with the following factors:

$$R\,i\,s\,k\,s\,c\,o\,r\,e=-665^{*}\,C\,D\,4+T\,c\,e\,l\,l\,s+6.2^{*}\,L\,E\,P+10.7^{*}\,N\,O\,X\,4$$

$$+-5.92^{*}RETNLB+-34.9^{*}LAIR1+5.67^{*}IL17A+-32.1$$

H*SPD1+23*CYTIP+-16.3*SLAMF7+-15.3*CD14

$$+-3.37^{*}C7+-19.5^{*}CORO2A+14.5^{*}PPARGC1B+$$

$$5.91^{*}LTB4R+4.7^{*}CHGA+17.5^{*}CD300A+-15.2^{*}TLR6$$

$$+16.8^{*}CD209+-14.5^{*}P2RY14$$

**FIGURE 6:**
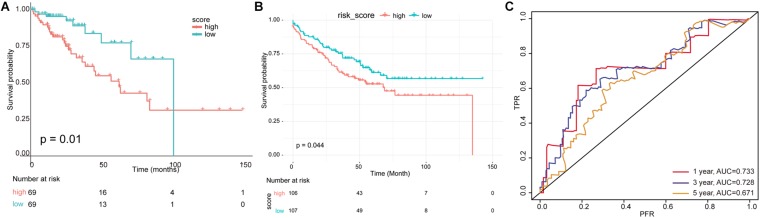
Evaluation of multivariate prognostic model. **(A)** A risk model was constructed using 19 immune-related factors, and the test set was divided into high- and low-risk groups according to scoring values. **(B)** The survival analysis for independent validation dataset. **(C)** The receiver operating characteristic (ROC) curve and area under the curve (AUC) value of survival time were predicted by multivariate risk model.

According to this risk score, samples in the test set were divided into high- and low-risk groups, and K–M survival analysis showed that the risk score has significant relevance with prognosis. The survival time of patients in the low-risk group was much longer than those in the high-risk group (*p* ≤ 0.01) in the TCGA test set (Figure 6A) and the independent validation dataset (Figure 6B). The AUC values of the survival time (1-/3-/5-year survival rate) in TCGA test set predicted by this risk model were 0.733, 0.728, and 0.711, respectively (Figure 6C).

### Nomogram Visualized Prognostic Model

**FIGURE 7:**
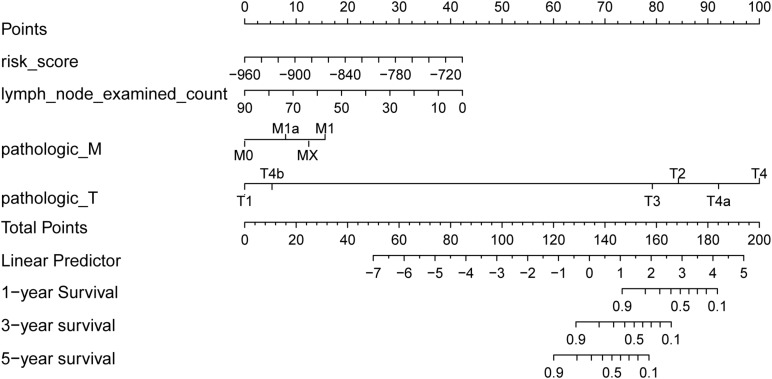
Survival rate predicted by nomograms. The factors used to construct the nomograms include risk score, lymph node examined count, pathologic M, and pathologic T. Total points are generated according to the values of these factors, corresponding to the survival probability of 1/3/5 years.

There were 127 samples with clinical number of lymph nodes and TNM stage in the test set. Based on the risk score of the risk model of multivariate Cox regression and prognostic clinical factors (lymph node examined count, pathologic M, and pathologic T), we visualized the nomogram of 127 test samples to show the risk model, as in Figure 7.

The results of the four prognostic factors in the nomogram and the *C* index and *p*-value of combined model for the Coxph model are shown in Table 2. The results showed that the *C* index of the combined model was the highest, and the risk score (*C* index > 0.7) had high fitting degree for the Coxph model. Among the clinical factors, only pathologic M had the most significant statistical test result (*p* = 0.0108).

**TABLE 2:** Analysis of prognostic factors to the fitting degree of Coxph model.

**Factor**	***C* index**	***p*-value**
Combined model	0.828	3.22*E*−21
Risk score	0.725	4.16*E*−11
Lymph node examined count	0.587	0.0956
Pathologic M	0.636	0.0108
Pathologic T	0.596	0.0647

## Discussion

In the tumor stroma, there exists a complex biological process between immune cells and malignant cells, and the immune system plays a dual role of promoting and inhibiting tumor growth, which is of great significance for prognosis (Nagalla et al., 2013). In this study, we identified hundreds of immune genes and analyzed the tumor-infiltrating immune cells in colon cancer. Based on these immune genes and immune cells, we constructed and validated a colon cancer diagnostic prediction model and a prognostic model. B cell infiltration ratio, *TRIB3*, *CHGA*, *CASP7*, and multiple LGALS family genes such as *LGALS4* were important features in diagnostic prediction model. Additionally, 19 factors such as CD4+ T cells, *LEP*, *NOX4*, *IL17A*, *HSPD1*, and *CHGA* had great influence on prognostic model.

In colorectal cancer, immune cells have significant infiltration, and their distribution, tissue localization, and cell types are highly relevant with progression and survival (Huh et al., 2012). Additionally, a study has reported that high infiltration of tumor-infiltrating immune cells in rectal cancer biopsies is related to improved tumor response to preoperative radiotherapy and chemotherapy, also with prolonged disease-free survival and OS (Anitei et al., 2014). In this study, B cell infiltration was found to be a diagnostic predictive feature of colon cancer, and CD4^+^ T cell infiltration was highly relevant with prognosis of colon cancer. A previous study has reported that infiltration of CD4^+^ lymphocytes is frequent in colorectal cancer (Diederichsen et al., 2003). Our results further provided evidence that immune cell infiltration may represent a favorable prognostic factor of colon cancer.

In addition to immune cells, we also identified some diagnostic prediction-associated immune genes, such as *TRIB3*, *CHGA*, *LGALS4*, and *CASP7*. Tribbles pseudokinase 3 (*TRIB3*) is upregulated in some colorectal tumors and is responsible for poor outcome. TRIB3 can interact with β-catenin and transcription factor 4 in intestine cells to increase expression of genes that are relevant with cancer stem cells in colorectal cancer (Hua et al., 2019). Human chromogranin-A (CHGA) is a 439-residue-long protein found in the secretory granules of some normal and neoplastic neuroendocrine cells, the expression of which is related to the prognosis of colorectal cancer (Gunay et al., 2019). Long and Campbell (2017) reported that male patients with low expression of galactin 4 (*LGALS4*) had significantly shortened disease-free survival in colon cancer. Caspase-7 (CASP7) plays an important role in the autophagy and apoptosis of colon cancer (Athamneh et al., 2017). Additionally, *CASP7* polymorphism is highly responsible for poor outcomes in patients with surgically resected colorectal cancer (Chae et al., 2011). Taken together, these genes may serve as diagnostic markers of colon cancer.

In this study, except for CD4^+^ T cells, 18 important genes were identified in prognostic model, such as *LEP*, *NOX4*, *IL17A*, *HSPD1*, and *CHGA*. Leptin (LEP) is a cytokine produced by adipose tissue and plays a role in promoting tumorigenesis (Housa et al., 2006). A study has reported that *LEP* polymorphism is responsible for an increased risk of developing colorectal cancer (Partida-Pérez et al., 2010). NADPH oxidase 4 (NOX4) is a major source of reactive oxygen species production, which has been reported to involve tumorigenesis (Juhasz et al., 2009). A recent study demonstrated that overexpression of *NOX4* can promote tumor progression and predict poor prognosis in human colorectal cancer (Lin et al., 2017). IL17A is a cytokine that can be produced by some immune cells. It has been reported to play an important immunopathogenic role in inflammation-related colonic diseases (Korn et al., 2009). Previous studies have reported that overexpression of *IL17A* means poor survival in colorectal cancer patients (Liu et al., 2011; Tosolini et al., 2011). Heat shock protein family D member 1 (HSPD1), as a signaling molecule in the immune system, is dysregulated in various cancers (Davalieva et al., 2015; Jin et al., 2016). Li et al. (2017) recently suggested that HSPD1 can serve as potential biomarker for the detection of colon cancer. Given the roles of these immune genes in colon cancer, we suppose that the constructed prognostic model may predict the prognosis of colon cancer.

## Conclusion

In conclusion, our analysis constructed an immune-related prognostic model of colon cancer. B cell infiltration ratio, CD4^+^ T cells, as well as genes of *TRIB3*, *CHGA*, *CASP7*, *LGALS4*, *LEP*, *NOX4*, *IL17A*, and *HSPD1* may be highly relevant with clinical outcome of colon cancer. Our results may help to uncover the clinical and biological significance of the immune microenvironment for colon cancer.

## Materials and Methods

### Gene Expression Data Collection

The colon cancer gene expression profile data for the TCGA cohorts were downloaded from the University of California Santa Cruz (UCSC) Xena database (Crawshaw et al., 2007), which included gene expression data of 329 samples (288 tumor samples and 41 normal samples). The log2(*x* + 1)-transformed RNA-Seq by Expectation–Maximization (RSEM) normalized read counts from UCSC Xena were downloaded for analysis. In addition, we also downloaded (download time: May 2019) the clinical phenotype data of the samples, including age, gender, TNM staging, and corresponding survival time and survival status. According to the gene annotation in the InnateDB database (Ge et al., 2002; Lang et al., 2016), 952 immune-related genes were annotated from the gene expression profile.

For the validation dataset, gene expression dataset of GSE17538 (Smith et al., 2010) was downloaded from Gene Expression Omnibus (Barrett et al., 2013). This dataset included gene expression data of 244 colon cancer samples. After deleting the samples without survival information, 213 colon cancer samples and the corresponding survival information were obtained. The gene expression data have been preprocessed by robust multiarray average (RMA) normalization in affy package. We downloaded the normalized gene expression data for further analysis.

### Differential Expression Analysis of Immune Genes

For immune genes, the differential expression analysis for tumor vs. normal samples was performed using the limma package (Bradizza et al., 2006) (version 3.10.3). The genes with zero or missing values were removed. After statistical test, we obtained the corresponding *p*-values of all genes. Benjamini and Hochberg method was used for multiple test correction to obtain the adjusted *p*-value (adj. *p*-value). The genes with adj. *p*-value < 0.05 and |log_2_FC| > 1 were selected as differentially expressed genes.

### EPIC Immune Cell Components Analysis

Based on the gene expression profiles of 329 TCGA samples, we used immune cell infiltration analysis tools EPIC^1^ (Racle et al., 2017) to analyze the immune cells ratio in each sample, including B cells, CAFs, CD4^+^ T cells, CD8+ T cells, endothelial cells, macrophages, NK cells, and other types of cells. For the infiltration ratio of each type of immune cells, Welch two-sample non-paired *t*-test was used to test the differences between tumor and normal samples statistically.

### Diagnostic Model of Colon Cancer Based on Immune Characteristics

The machine learning model was constructed in light of the immune cell components and differentially expressed gene information. Based on the integrated learning methods of GBDT, XGBoost, RandomForest, as well as the classification models of SVM and LR in Sklearn database^2^, we used the GridSearchCV package to build a model and adjust preferences. After adjustment and comparison of different models, we constructed an effective training classifier, which could predict colon cancer based on gene expression values. Here, we used the preprocessing.scale method to normalize the samples. Then, the samples were randomly divided into training and test sets at a ratio of 6:4 using train_test_split model from Sklearn database (random state = 123). We used cross-validation to validate the training model and added the parameter of class_weight = “balanced” during the training to eliminate the influence of category imbalance. Finally, model evaluation was carried out on test set based on accuracy and AUC (mainly based on AUC value).

### Gene Expression/Clinical Phenotype and Prognosis Analysis

The prognosis information of corresponding patients was collected on the basis of the downloaded clinical data, including OS and OS status. Combining the differentially expressed immune genes and patient’s clinical phenotype, which are the candidate features with the sample prognostic information, we conducted the Kaplan–Meier (K–M) survival analysis by dividing the patients into high and low-expression groups based on gene expression or phenotypes. We used log-rank test to calculate the *p*-value and preliminarily screened genes with *p* < 0.05 to obtain genes and clinical phenotypes associated with prognosis.

### Prognostic Model of Colon Cancer Based on Immune Characteristics

The TCGA data set was randomly divided into training and test sets at a ratio of 5:5 (random state = 123). In the training set, we used the prognostic immune cell components and immune gene expression, as well as the Coxph method in R package “survival” to comprehensively consider the effects of these characteristics and obtain the correlation significance [Pr(>|*z*|)] and coefficient (coef) of each feature. Here, we screened the genes with significance *p* < 0.05. We used the screened significant genes and constructed the following multivariate risk model:

Riskscore=βgene1×exprgene1+βgene2×

exprgene2+…+β⁢geneN×exprgene

where βgene1, βgene2,…β,geneN represents the coefficient of each gene in a multifactor analysis.

For each sample in TCGA test set and the validation dataset, we calculated the risk score according to this formula and divided the samples into high- and low-risk groups on the basis of the median value of risk score. Then, K–M survival analysis was performed. In addition, we used this risk score as the standard to predict the 1-/3-/5-year survival rate of patients in TCGA test set, and the receiver operating curve (ROC) and AUC value were used for model evaluation.

### Validation of the Prognostic Model in Independent Dataset

The EPIC was used to analyze the immune cells ratio in each sample of GSE17538, including B cells, CAFs, CD4^+^ T cells, CD8^+^ T cells, endothelial cells, macrophages, NK cells, and other types of cells. Risk score of each sample in validation dataset was calculated based on the following formula:

$$R\,i\,s\,k\,s\,c\,o\,r\,e=-665^{*}\,C\,D\,4+T\,c\,e\,l\,l\,s+6.2^{*}\,L\,E\,P+10.7^{*}\,N\,O\,X\,4$$

$$+-5.92^{*}RETNLB+-34.9^{*}LAIR1+5.67^{*}IL17A+-32.1$$

H*SPD1+23*CYTIP+-16.3*SLAMF7+-15.3*CD14

$$+-3.37^{*}C7+-19.5^{*}CORO2A+14.5^{*}PPARGC1B+5.91$$

L*TB4R+4.7*CHGA+17.5*CD300A+-15.2*TLR6

$$+16.8^{*}CD209+-14.5^{*}P2RY14$$

All samples were divided into high- and low-risk groups according to the median value of the risk score. K–M survival analysis and log-rank test were performed for samples in high- and low-risk groups.

### Nomogram Visualized Prognostic Model

The survival rate could finally be obtained by mapping different factors to points and then adding them together. Therefore, on the basis of prognostic model risk score and prognostic clinical phenotypes, the nomogram prediction model was constructed and visualized. In order to further verify the predictive power of nomogram, we first calculated the independent prognostic factors and composite factors in the nomogram to fit the consistency index (*C* index) of Coxph model. On top of that, resampling method was used to carry out statistical test to calculate the *p*-value and compare the fitting degree of each independent prognostic and composite factors to the Coxph model.

## Data Availability Statement

The raw data supporting the conclusion of this article will be made available by the authors, without undue reservation, to any qualified researcher.

## Author Contributions

All authors listed have made a substantial, direct and intellectual contribution to the work, and approved it for publication.

## Conflict of Interest

The authors declare that the research was conducted in the absence of any commercial or financial relationships that could be construed as a potential conflict of interest.

## Footnotes

1 https://gfellerlab.shinyapps.io/EPIC_1-1/

2 https://scikit-learn.org/
